# Supplementary material for: Multifunctional aggregation network of cell nuclei segmentation aiming histopathological diagnosis assistance: A new MA-Net construction
Source: PLoS One. 2024 Sep 6;19(9):e0308326. doi: 10.1371/journal.pone.0308326 (PMC11379384; doi:10.1371/journal.pone.0308326)
Supplement: Attachment — Submitted filename: Review Comments_PONE-D-24-00318.docx [file pone.0308326.s001.docx]

**Ref.:  Ms. No. PONE-D-24-00318
Multifunctional Aggregation Network of Cell Nuclei Segmentation Aiming Histopathological Diagnosis Assistance: A New MA-Net Construction**

The paper introduces a method U-Net based model, MA-Net(Multifunctional Aggregation Network), to accurately segmenting nuclei and the model utilized the subsampling fusion module and upsampling fusion module on the U-Net encoder and decoder respectivel. Overall, the manuscript has a good structure and better validation of results via ablation studies. However, I suggest the manuscript to undergo major revision and resubmit for another quick review.

The following are some major corrections that can be incorporated to improve quality:

1) More experiments on several other datasets such as Fluorescence Microscopy Image Dataset and any other medical image segmentation datasets has to be performed to effectively understand the contribution of the proposed approach and validation of methodology. I suggest authors to make more evaluation on similar datasets.
2) Similarly, a detailed comparison on several recent methods (papers published on or after 2020) such as TransNuSeg, CellViT, has to be performed. The Authors can refer to <https://paperswithcode.com/sota/medical-image-segmentation-on-monuseg> for more details on the state-of-the-art methods.
3) The Abstract is too long and can be condensed highlighting the novelty and best results on datasets.
4) The recommend authors to highlight the novelty in methodology. The manuscript is very similar to CE-Net (Context Encoder Network for 2D Medical Image Segmentation) thereby might be a further extension to cell nuclei segmentation application.
5) I suggest authors to add future scope for further improvements and research in this direction and add and explain a figure for some of the failure cases of using this methodology.

Minor corrections:

1) Extra full stop on the first line in Abstract. I recommend Authors to carefully read and review the manuscript for any typographical errors.
2) Equations can be enhanced in the methodology. I suggest authors to use LaTeX while formulation equations.
3) Hyperlinks has to be added to citations and references for helping readers to quickly refere the cited studies.
