# Supplementary material for: Multifunctional aggregation network of cell nuclei segmentation aiming histopathological diagnosis assistance: A new MA-Net construction
Source: PLoS One. 2024 Sep 6;19(9):e0308326. doi: 10.1371/journal.pone.0308326 (PMC11379384; doi:10.1371/journal.pone.0308326)
Supplement: Attachment — Submitted filename: Response to Reviewer Comments.docx [file pone.0308326.s002.docx]

**Cover Letter**

**PONE-D-24-00318R1**

***Plos One***

Dear Editor,

Thank you for your serious and constructive comments on our manuscript. All your and reviewers’ suggestions are very important. According to these suggestions, the manuscript has been revised with the highlight in blue for the modifications. Moreover, we have responded all comments from reviewers point-by-point in the following letter. We hope that the revised manuscript can be acceptable in Journal of *Plos One*.

Thank you and best regards

Yours sincerely,

Corresponding author:

Name: Qiumei Pu Ph.D., Associate Professor; Lina Zhao, Professor

Address: Minzu University of China, 27 Zhongguancun South Avenue Beijing, Beijing 100081, P. R. China; CAS Key Laboratory for Biomedical Effects of Nanomaterials and Nanosafety Institute of High Energy Physics, Chinese Academy of Sciences

Tel: 86-10-68932970

Fax: 86-10-68932970

Email: [puqiumei@muc.edu.cn;](mailto:puqiumei@muc.edu.cn;) [linazhao@ihep.ac.cn](mailto:linazhao@ihep.ac.cn)

**Response to reviewers**

**Major Corrections**

**1.Comment:** More experiments on several other datasets such as Fluorescence Microscopy Image Dataset and any other medical image segmentation datasets has to be performed to effectively understand the contribution of the proposed approach and validation of methodology. I suggest authors to make more evaluation on similar datasets.

**Reply:**

Thank you for your suggestions. We have conducted additional experiments using more datasets to further demonstrate the effectiveness of our proposed method. The results and analysis of these experiments have been added to Section 4.3 "Results and Discussion" in the main text.

**2.Comment:** Similarly, a detailed comparison on several recent methods (papers published on or after 2020) such as TransNuSeg, CellViT, has to be performed. The Authors can refer to https://paperswithcode.com/sota/medical-image-segmentation-on-monuseg for more details on the state-of-the-art methods.

**Reply:**

Thank you for your suggestion. To further validate the effectiveness of our proposed method, we conducted additional experiments using state-of-the-art Transformer-based approaches post-2023 on the MoNuSeg and TNBC datasets. The results and analysis of these experiments are presented in Section 4.3, Results and Discussion.

**3.Comment:** The Abstract is too long and can be condensed highlighting the novelty and best results on datasets.

**Reply:**

Thank you for reviewing our paper and providing your suggestions. We understand that the abstract is an essential part for readers to quickly grasp the research content and outcomes. We have made the necessary simplifications and optimizations to the abstract in the revised manuscript.

**4.Comment:** The recommend authors to highlight the novelty in methodology. The manuscript is very similar to CE-Net (Context Encoder Network for 2D Medical Image Segmentation) thereby might be a further extension to cell nuclei segmentation application.

**Reply:**

Thank you for reviewing our paper and for your valuable comments. While both our work and CE-Net focus on semantic image segmentation, and thus inevitably share some similarities, it is important to emphasize that our work is not an extension or continuation of CE-Net. Our design approach has several key differences:

Firstly, CE-Net's improvements mainly focus on the Bottleneck part of the U-Net network, whereas our work involves improvements to the encoder, decoder, skip connections, and Bottleneck of the U-Net. We conducted comparative experiments using CE-Net as a baseline model, and the results demonstrate the effectiveness of our proposed feature fusion strategy and spatial attention mechanism. Secondly, our work differs from CE-Net in terms of training setup. Considering the characteristics of cell nucleus medical imaging, we adopted a hybrid loss function different from CE-Net and employed different data augmentation methods. Lastly, in terms of application, CE-Net is applied to retinal vessel images, while our work primarily targets cell nucleus segmentation.

Regarding your concerns about the novelty of our method, we have revised the abstract and relevant sections of the main text to further emphasize the originality and novelty of our work.

**5.Comment:** I suggest authors to add future scope for further improvements and research in this direction and add and explain a figure for some of the failure cases of using this methodology.

**Reply:**

Thank you for your suggestions. We also recognize the importance of analyzing the current limitations and considering improvements for future work. We have added relevant content at the end of Section 4.3 "Results and Discussion" and in Section 5 "Conclusions".

**Minor Corrections**

**1.Comment:** Extra full stop on the first line in Abstract. I recommend Authors to carefully read and review the manuscript for any typographical errors.

**Reply:**

Thank you for pointing out the issues. We have carefully reviewed the manuscript of the article and made corrections to the formatting and textual errors.

**2.Comment:** Equations can be enhanced in the methodology. I suggest authors to use LaTeX while formulation equations.

**Reply:**

Thank you for your suggestions. We have carefully reviewed and verified the formulas in the text, and we have used MathType to rewrite them, correcting any formatting and layout errors.

**3.Comment:** Hyperlinks has to be added to citations and references for helping readers to quickly refere the cited studies.

**Reply:**

Thank you for your suggestion. We have added hyperlinks to all the references cited in the article. Readers can now click on the citation numbers in the text to navigate to the corresponding references.

Special thanks to you for your good comments.
